# The Dental Protective Association

**Published:** 1890-08

**Authors:** J. N. Crouse


J. N. Crouse addressed the Association on

### The Dental Protective Association.

*Mr. President and Members of the Michigan State Dental Association:*

When I appear before a body of men like this, to address them in the interest of the Dental Protective Association, I don't know where to begin. I have been laboring earnestly for some time in the interest of the Dental Protective Association, an organization whose principal aim is to bind the dental profession together for mutual protection against the unjust demands of parties wishing to make us divide part of our earnings without any legal claim.

I am here today to find out the reason why of the six hundred dentists practicing in the State of Michigan, only twenty-five have joined the Dental Protective Association. We have issued and mailed to every dentist in this State whose address we could obtain two carefully prepared circulars, presenting the object and aims of the Dental Protective Association, and I am compelled to believe that not one man in twenty has read those circulars carefully or we should have more than twenty-five members from this State.

I come to you with the conviction that you are not aware of the trouble and worry that awaits you if you do not take steps immediately to protect yourselves from the unjust demands of one of the most abominable monopolies that ever threatened the welfare of the dental profession.

I want to tell you something about the International Tooth Crown Co. which claims a patent on almost every process that a dentist uses. This is not the only adversary we have threatening us. This company claims patents on all kinds of bridge-work, both permanent and removable, patents on cutting off crowns of natural teeth, freezing the tooth and destroying the pulp and driving it out with a wedge of wood, patents on filling the end of the root, and on material for setting a pin or screw in the root to hold the crown or bridge, its patents cover almost every form of crown made, those with as well as without bands, it even has patents on taking impressions, methods for getting the articulation, investing for soldering. In fact it patents everything it can and buys every patent it can get; it makes no difference whether the patent is a valid one or not, as an individual dentist can not afford to contest its validity, hence the necessity of combining in an association.

But I am here today to tell you that the Dental Protective Association, on the advice of its attorneys, the best patent lawyers in the United States, is prepared to defend its members against any patent claim of this company, if furnished with the money necessary to carry on the litigation. This may appear to be a strong statement, but members of the old rubber company assure us that if the dental profession had been organized, as they now are, this company would not have been successful in collecting the eight or ten million dollars it did. The ten thousand dentists of the United States with a fund of $100,000, and organized to protect themselves against these unjust demands, will command respect, and be able to collect evidence, employ first-class attorneys and make an adequate defence, such as no individual could possibly do.

I tell you if it were not for the Dental Protective Association, there would not be more than ten men in this room or in the dental profession in this country that would not be paying a royalty on the meanest patents owned by the International Tooth Crown Co.

It is an easy matter to get a patent on almost anything that is not actually covered by some patent. If there is no interested person to look after this matter what is to prevent the wholesale patenting of every useful device or process now used or to be discovered in the future, and so check the progress of our profession? The Dental Protective Association will not interfere with the granting of lawful patents, but it means to stand between the dental profession and all illegal patents.

How can you help the Dental Protective Association in this work? Don't take out a license from the International Tooth

Crown Co. If you do you are compelled not only to recognize all its present patents as valid, but all it may in the future secure or forfeit as a penalty $25. If a contract of that nature can be made strong enough to stand, and besides all the money you pay into this corporation makes it that much stronger and enables it to continue its fight with the Protective Association. Send ten dollars to the Association and become one of us, and we will defend your suit for you. The Tooth Crown Company has not sued a member of the Association, it is the man who is not able to make a sufficient defense that will be sued or bluffed into taking a license. The Crown Co. now has a suit in the Supreme Court involving the Richmond crowns, cutting off teeth, extirpating pulps, etc., which we expect they will win, for the reason that at the time the suit was tried, those trying it had not the evidence that we now possess, and which we can not use in this case. Should this case be won by the Crown Co. it will not discourage the Protective Association or affect the interest of our members, for we have evidence that will be used in new suits which will surely win.

Send us your money and help us to gather evidence by sending us a record of any crown or bridge-work known to have been done prior to 1881. Thus you will help to fortify us by putting in our hands the means for defending you, and your peace of mind will be secured at a very small outlay of money. Don't hold on to your money but give it up cheerfully. We will not defend the dental profession, but we will defend the members of the Dental Protective Association.

DISCUSSION.

Dr. J. E. Low, of Chicago, wanted to know of Dr. Crouse what was the attitude of the Dental Protective Association toward patents in general?

Dr. Crouse: The Dental Protective Association has no fight with legitimate patents or with men who invent, own or honorably dispose of their patents. If I was smart enough to invent a good thing I should patent it and sell it too if I could, but I would do it honestly.

Dr. George L. Field, of Detroit: If the Gaylord suit now pending before the Supreme Court should be decided in favor of the International Tooth Crown Co., it will go hard with the dentists. But there is no fear that the Dental Protective Association will not be able to protect its members at any rate. I would not pay any money to nullify a legitimate patent owned by an individual, but I do object to being bled by a corporation that doesn't care how or where it gets a patent, or what the value of the patents it controls may be so long as they can be used to extort money unjustly from men unable to defend themselves. The old Rubber Co. by secret and underhand methods secured a judgment against me for $20,000, and the sheriff had levied on my property before I was aware of it, and we may look for just such underhand practices in this case. The Dental Protective Association is organized for the purpose and can take care of your suits better than you can possibly do and I advise every man here to join it at once.

Dr. Land, of Detroit, asked the privilege of the association to state that he was the author of sixteen patents which he held in the interest of the dental profession, and he was prepared to contribute his ten dollars to the Dental Protective Association as an indication of his approval of its object.

Dr. A. M. Long: It is a matter of interest and commendation that Dr. Land has not sold any of his sixteen patents, as I know he has had many very good offers to dispose of them advantageously.

Dr. J. Taft: It seems to me that here is an opportunity for co-operation on the part of the dental profession that should not be lost sight of. The banding together of such a large number in the interest of a common cause will have the effect of bringing the dental profession into more harmonious relations. I do not object to legitimate patents or to traffic in them, but I can not indorse the methods of the International Tooth Crown Co. It is a surprise to me that so few in this State have joined the Dental Protective Association. I hope every one here will feel that it is not only his privilege but his duty to join the association and so indorse its objects and methods and encourage those who are giving their time to its work.

Dr. W. H. Dorrance, Ann Arbor: I feel that Dr. Crouse has done a grand work for the dental profession by taking up this cause, and I feel that we should sustain him with our council, sympathy, and money. I hope every man here will feel inclined to join the Dental Protective Association and help to carry this war to a successful termination.

Dr. G. E. Corbin, St. Johns: I would like to ask whether there is, or can be any further liability of members of the Dental Protective Association beyond the fee of ten dollars required for membership? Also, if a member of the Dental Protective Association is sued by the Tooth Crown Co., and after being defended by the association and a judgment is rendered against him, will the Protective Association pay, or the defendant? How much could the company sue for?

Dr. Crouse: The members of the association may be called upon, by such provision in its constitution, for an additional ten dollars only, and this only in case of extreme need. It is not likely any member will ever be called on for this extra assessment. When a member is sued the association takes entire charge of the defense, pays all costs of court and lawyers' fees, but does not agree to pay the judgment should one be obtained against the defendant. But this need not frighten any one, for by United States law the judgment can not be for more than the royalty would have amounted to if paid as a license, so that no one can lose more than the ten dollars he puts into the association. Gentlemen, if you will believe me you will never regret the money you put into this work. You don't want some one else to do the work and pay the cost of doing a thing that concerns you just as much as it does any one else. Pay your share and thus do your share, and you will be able to rejoice with us in this grand work when it is finished.

Dr. Sanders, Saginaw: I had several calls from the agent of the Tooth Crown Co. and received several letters from the company with invitations to take out a license, but I concluded to join the Protective Association and I have not heard from the Crown Co. since.

Dr. Douglas moved the appointment of a committee to frame a resolution expressing the attitude of the Michigan Dental Association toward the Dental Protective Association. The motion carried and the president appointed Drs. Dorrance, Douglas and Sanders, the committee reported the following resolutions which were unanimously adopted:

*Resolved*, That the Michigan Dental Association heartily approves the aim and plan of the Dental Protective Association; and that it is further

*Resolved*, That it is the duty of every member of the dental profession in this State to join the Dental Protective Association.

It is also *Resolved*, That Dr. Crouse be requested to furnish the dental journals with an abstract of his remarks for publication.

W. H. DORRANCE,
W. D. SANDERS,
E. G. DOUGLAS, Committee.

---

The following officers were elected for the ensuing year: President, H. K. Lathrop; 1st Vice-President, H. C. Corns; 2nd Vice-President, F. S. Owen; Secretary, J. Ward House; Treasurer, Geo. H. Mosher; Board of Censors, J. L. Gish.

By a unanimous vote it was decided to hold the next meeting the third Tuesday in August, 1891, at the Sault Ste Marie.

On the recommendation of the Publication Committee the papers read before the Society were orded to be given to the DENTAL REGISTER and the *Ohio Journal of Dental Science* for publication, with a report of the proceedings.

On motion, adjourned to 3 P. M.

Meeting called to order at 3:30 P. M., after a very interesting visit to the State Prison where Warden Hatch received the association cordially, and after visiting the workshops and seeing the men at work, addressed them on the subject of prison reform. The address was exceedingly interesting and all felt that the State of Michigan was exceedingly fortunate in having so excellent a man at the head of this great institution. A rousing vote of thanks was cheerfully given Mr. Hatch, for his courtesy.

A vote of thanks was extended to Mr. D. C. Meseroll, of Jackson, for the beautiful programmes he furnished the association. A vote of thanks was also given to the common council of Jackson, for the use of the Council Chamber.

On motion, twenty-five dollars was ordered to be paid to the secretary for services rendered. On motion, N. S. Hoff, of Ann Arbor, G. E. Sanders, of Saginaw, and L. D. Wood, of Grand Rapids, were appointed a committee to solicit members for the Dental Protective Association.

The Board of Directors reported the following bills of expense, which were ordered paid: To the janitor, $6.00; The Citizens Printing House, $15.30; John L. Gish, $13.59; George H. Mosher, $12.00; Wm. Cleland, 3.00.

The treasurer made the following report, which was received and adopted:

| | |
|---|---|
| Balance on hand from 1889 | $104.45. |
| Received at present session | $150.00. |
| Total, | $254.45. |
| Disbursements present session | $45.89. |
| Balance on hand, | $204.56. |

The president appointed Dr. E. C. Moore, of Detroit, a member of the Executive Committee; Drs. E. H. Conway and W. L. Williams, of Sault Ste Marie, as Local Committee of Arrangements; Drs. C. S. Case, Wm. Cleland, and J. Ward House, as Publication Committee; Dr. Geo. L. Field, University Visiting Committee.

On motion, adjourned. WM. CLELAND, Sec.

---

THE next meeting of the National Association of Dental Examiners will be held in Excelsior Springs, Mo., on Monday evening, August 4th, at 8 o'clock, and at other times during the week, between the sessions of the American Dental Association. It is important to have every State Board represented.

FRED. A. LEVY, D.D.S., Secretary.

---

IT is claimed that warts are easily removed by applying a drop or two of a saturated solution of carbonate of soda, four or five times a day.

